# Comparing the Efficacy of 8.4% and 7.5% Buffered Lidocaine during Surgical Removal of Impacted Mandibular Third Molar- A Split Mouth Double Blinded Randomized Controlled Study

**DOI:** 10.30476/dentjods.2023.98891.2115

**Published:** 2024-09-01

**Authors:** Balamurugan Rajendran, Surabhi R Jain, Jane Belinda Tony

**Affiliations:** 1 Oral and Maxillofacial Surgeon and Oral Implantologist, RYA COSMO Foundation Hospital, Chennai, India; 2 Dental Surgeon, RYA COSMO Foundation Hospital, Chennai, India; 3 Periodontist and Oral Implantologist, RYA COSMO Foundation Hospital, Chennai, India

**Keywords:** Tooth, Impacted, Lidocaine, Pain

## Abstract

**Statement of the Problem:**

Conventional injection technique with adrenaline during removal of impacted third molar of mandible had proportionally increased pain during administration with slow onset of action and shorter duration of anesthesia.

**Purpose:**

The purpose of this study was to compare the effective nature of 8.4% and 7.5% buffered lidocaine hydrochloride during surgical removal of mandibular impacted third molar.

**Materials and Method:**

This prospective crossover study included 50 patients requiring bilateral removal of impacted mandibular third molars. Group I included 50 impacted mandibular third molars that were administered with 8.4% buffered lidocaine hydrochloride and group II included 50 impacted mandibular third molars were administered with 7.5% buffered lidocaine hydrochloride. The outcome variables were pain on injection, time of onset of anesthesia, and duration of action of anesthesia. The above parameters were recorded by the investigator and statistically analyzed through Chi-square test using SPSS software.

**Results:**

Patients in group I had mild pain (1.02) and patients in group II (5.74) had moderate pain with a statistical significance of *p*< 0.05 for group I respectively. The mean onset of action of anesthesia in group I was 0.08 seconds and 0.59 seconds in group II (*p*< 0.05). The duration of anesthesia was 342.51 minutes from group I and 122.06 minutes in group II (*p*< 0.05) respectively.

**Conclusion:**

Lidocaine hydrochloride buffered with 8.4 % sodium bicarbonate was found to be more effective in reduction of pain during injection, also had a faster onset of action and longer duration of the action of anesthesia when compared to 7.5% buffered lidocaine hydrochloride.

## Introduction

Local anesthesia poses a wide range of its use in the field of dentistry for adequate control of pain producing long-term blockade of the sensory nerve and subsequently minimizes the requirement of analgesics after any oral surgical procedures. The predominant use of local anesthesia being 2% lidocaine hydrochloride with 1 in 80,000 concentrations of adrenaline at a pH ranging from 3.3-5.5 [ 1 - 3 ]. The local anesthesia formulated at a low pH increases the solubility and prolongs the shelf life of the solution, which in turn prevents the adrenaline oxidation causing burning sensation on the injected tissue site [ 1 ].

An increase in the pH of local anesthesia fastens the action and efficacy of anesthesia providing comfortable injection experience to the patients. Alkalinization of local anesthesia can be prepared by adding sodium bicarbonate [ 4 ]. This eventually increases the free base form of lidocaine and alkalinizes the solution reducing pain during administration [ 5 - 6 ]. Extensive studies have been dealt with the use of sodium bicarbonate at different concentrations of 7.5% and 8.4% in comparison with 2% lidocaine hydrochloride and their successive outcome measures were significantly evaluated on a larger scale [ 3 , 7 - 8 ].

The present study aimed to evaluate the efficacy of two different concentrations of buffered lidocaine 8.4% and 7.5% on pain during administration of anesthesia, the onset of anesthesia, and duration of the action of anesthesia during surgical removal of mandibular impacted third molar.

## Materials and Method

### Study Design and Enrolment

This prospective split mouth double blind randomized controlled study was conducted on 100 impacted mandibular third molars of 50 patients between age groups of 18-35 years, who reported to the Department of Dentistry, RYA COSMO Foundation, Chennai, India for surgical extraction of bilateral impacted mandibular third molars. The present study was performed based on the Consort Statement Guidelines 2010. The study proposal was reviewed and approved by the RYA COSMO Foundation, EC/RYA/006. Informed consent was obtained from all the study participants included in this study. Patient inclusions were defined as age groups between 18-35 years, bilateral impacted mandibular third molars, ASA category I and II, and patients without any signs and symptoms of infection or inflammation during the procedure. Exclusion criteria were defined as unilateral impacted mandibular third molars, ASA category III and IV, pregnant and lactating patients, patients with a history of any systemic diseases, and patients allergic to medications.

### Determination of sample size

The sample size for the current study was estimated using SPSS software G* power 3.1.92. The effect size was set at 0.32 with ∝ error 0.05 and power 95% was determined to be 100 impacted mandibular third molars (50 in each group).

### Randomization

Administration of 8.4% and 7.5% buffered lidocaine hydrochloride was randomly assigned between groups through a simple random sampling using lottery method.

### Blinding

Two syringes were taken one containing 8.4% buffered lidocaine hydrochloride and other syringe containing 7.5% buffered lidocaine hydrochloride was covered with numbers 1 and 2. This was done to blind the patient and the operating surgeon. The investigator was the only person aware of the local anesthesia administered to each patient during the study. 

### Groups

The group I consists of 50 impacted mandibular third molars that were administered with 8.4% buffered lidocaine hydrochloride, and group II included 50 impacted mandibular third molars administered with 7.5% buffered lidocaine hydrochloride. In the first appointment, the local anesthesia from syringe 1 was administered. Later, the patients were followed up after 10 days for the removal of contralateral tooth. During the second appointment, the solution from syringe 2 was then administered and vice-versa. None of the participants was lost for the follow up.

### Materials required

8.4% sodium bicarbonate (SODAC, Neon Laboratories Limited, Mumbai, India), 7.5% sodium bicarbonate (sodium bicarbonate, Hindustan Chemicals and Pharmaceuticals, Mumbai, India), 2% lidocaine hydrochloride with 1:80000 concentration adrenaline (Lignox®2% A, Indoco Remedies Ltd, Maharashtra, India)

### Preparation of 8.4% and 7.5% buffered lidocaine hydrochloride

**Step 1:** 0.6ml of sodium bicarbonate was drawn from a 20ml ampule of 8.4% NaHCO_3_ W/V, 50mEq/50ml in one syringe and 7.5% NaHCO_3_ W/V, 50mEq/50ml in other syringe [ 8 ].

**Step 2:** The above solution was added to 3ml of 2% lidocaine hydrochloride with 1: 80,000 concentration of adrenaline in a 5ml syringe.

**Step 3:** The final concentration of 0.18mEq/ml with 8.4% sodium bicarbonate and 0.17mEq/ml with 7.5% sodium bicarbonate was obtained. 

**Step 4:** The loaded syringe was then mixed thoroughly and finally checked for precipitation. The solution must be free from particulates or cloudiness.

### Surgical Procedure

The removal of impacted mandibular third molar was performed by the same oral surgeon. The Inferior alveolar nerve block was administered based on the study protocol for each group. Ward’s incision was placed using #15 blade and triangular flap was raised using Molt’s periosteal elevator to expose the underlying impacted tooth and the bone. Mesial, buccal and distal bone guttering was done using #702 bur and the tooth was sectioned or removed completely through elevators and forceps. The peripheral bony margins were smoothened and the extracted socket was irrigated with povidone iodine. The mucoperiosteal flap was then freshened, and the socket was sealed through primary closure. 

### Method of assessments

Patients were evaluated immediately following deposition of local anesthesia in both the groups. Pain during administration was assessed using 10 cm visual analogue scale (VAS). Time of onset of anesthesia was calculated as the time starting from the point of retrieval of the needle after injection till the first sensation of numbness or tingling in the anaesthetized region using a stopwatch. Duration of anesthesia was obtained by requesting the patients to inform, the moment the effect of local anesthetic wore off. 

### Statistical Analysis

The outcome variables obtained between groups were prepared in a standard proforma by the investigator and the recorded data was calculated using SPSS software, 14.0version, Chicago, USA. The significant differences such as mean and standard deviation for each parameter were analyzed using descriptive statistics and the independent significant variables between groups were analyzed through chi-square test. P value less than 0.05 was considered significant for the present study.

## Results

**Figure 1:**
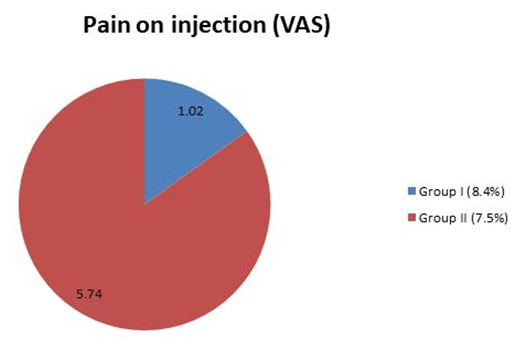
The average values between groups for pain on injection (visual analogue scale)

**Figure 2:**
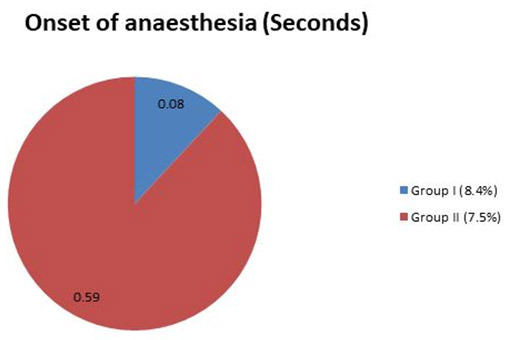
The average values between groups for onset of anesthesia (seconds)

**Figure 3:**
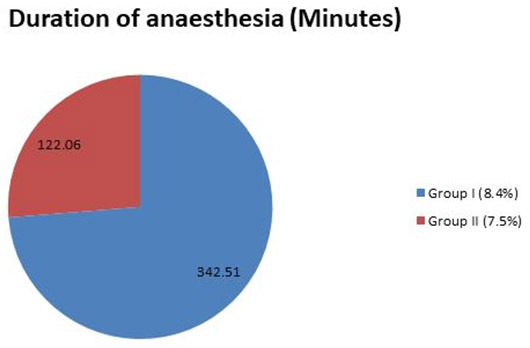
The average values between groups for duration of anesthesia (minutes)

A total of 100 impacted mandibular third molars in 50 patients were included as a crossover study. The study participants were recruited with an average age of 33 years, of whom 32 patients were males and 18 patients were females. The data obtained from the present study were statistically analyzed by a chi-square test using SPSS software (Table 1, Figures 1-3). 

**Table 1:** Chi-square test of significance between group I (8.4% buffered lidocaine) and group II (7.5% buffered lidocaine) on pain during injection, onset of action of anesthesia and duration of the action of anesthesia

Parameters	Group I (8.4%) Mean (SD)	Group II (7.5%) Mean (SD)	*p* Value <0.05
Pain on injection (visual analogue scale)	1.02(0.06)	5.74 (1.26)	0.04
Onset of anesthesia (seconds)	0.08(0.03)	0.59 (0.09)	0.02
Duration of anesthesia (minutes)	342.51(2.58)	122.06 (1.73)	0.01

### Comparison of outcome variables between group I and group II

### Pain during injection (vas)

All 50 patients in group I had only mild pain during administration of anesthesia whereas in group II, 38 patients had moderate pain and 12 patients had mild pain with a mean difference of 1.02 (0.06) for group I and 5.74 (1.26) for group II which showed a statistical significance of *p*< 0.05 for group I respectively.

### Onset of anesthesia (seconds)

The participants in group I had a faster onset of action (0-30 seconds) when compared to group II participants (30-60 seconds) with a mean difference of 0.08 (0.03) for group I and 0.59 (0.09) for group II which showed a statistical significance of *p*< 0.05 for group I respectively.

### Duration of anesthesia (minutes)

Group I participants had longer duration of anesthesia (300-420 minutes) when compared to participants in group II (60-240 minutes) with a mean difference of 342.51 (2.58) for group I and 122.06 (1.73) for group II which showed a statistical significance of *p*< 0.05 for group I respectively.

## Discussion

Local anesthetic solutions are generally formulated and manufactured with pH 3.9 to prolong the solubility and shelf life of anesthesia [ 9 ]. The pH of local anesthesia without vasoconstrictor was found to be 6.5 and by adding vasoconstrictor, the pH of the solution is further reduced to 3.8-5 [ 10 ]. The acidic nature of the solution when administered into the tissue produces pain and burning sensation [ 10 ]. This decreases the amount and rate of RN base molecules crossing the epineurium, simultaneously reducing the efficacy of anesthesia. The acidic form of local anesthesia can be alkalinized with the use of sodium bicarbonate. The local anesthesia when buffered with sodium bicarbonate had drastically reduced the pain during injection and also improved the anesthetic efficacy [ 11 ].

Numerous studies have demonstrated the utility of buffered local anesthesia in comparison with non-buffered local anesthesia and their nature of efficacy has been clearly dealt in English literature. The results of their studies suggested that, buffered lidocaine had a faster onset of action and longer duration of anesthesia when compared to non-buffered lidocaine [ 3 , 7 , 12 ]. However, no studies have proven the efficacious value by comparing two different concentrations (8.4% and 7.5%) of sodium bicarbonate. The present study has evaluated the anesthetic efficacy of 8.4% buffered lidocaine hydrochloride and 7.5% buffered lidocaine hydrochloride for pain during injection, onset of anesthesia and duration of anesthesia in the removal of impacted mandibular third molar. 

Fear of pain imparted to the patient during the administration of local anesthesia influences the further progression of the treatment [ 13 - 14 ]. Pain during deposition of local anesthesia predominantly depends on varying factors such as the speed of deposition of anesthesia, presence of any local inflammation at the site of injection, tissue tension during administration, and pH of anesthesia [ 10 ]. Local anesthesia when buffered with sodium bicarbonate reduces pain during injection in two possible ways. First, sodium bicarbonate increases the availability of pH in the solution similar to the physiologic pH, thereby providing comfortable administration of injection. Second, the number and rate of RN molecules increased, hence the duration of these molecules available within the tissues are relatively short [ 10 ]. 

In the recent study, all the 50 patients in group I (8.4%) had mild pain and in group II (7.5%), 12 patients had mild pain and 38 patients had moderate pain, while administration of local anesthesia with a mean difference of 1.02 in group I and 5.74 in group II which showed a statistical significance of *p*< 0.05 for group I. The above findings were similar to the results obtained by Younis *et al*. [ 15 ] and Ruegg *et al*. [ 16 ] where the participants included in their study reported with less pain on injection with buffered lidocaine. On contrary, Whitcomb *et al*. [ 17 ] and Chaney *et al*. [ 18 ] found no significant reduction in pain when injected with buffered lidocaine. 

The onset of action in the current study was in the range of 0-30 seconds in all 50 patients of group I (8.4%) and patients in group II (7.5%) had the onset of action in the range of 30-60 seconds with a mean difference of 0.08 seconds for group I and 0.59 seconds for group II, which showed a statistical significance of *p*< 0.05 for group I. The obtained results of our study had a positive correlation with Agarwal *et al*. [ 8 ], Christoph *et al*. [ 9 ], DiFazio *et al*. [ 19 ], Zahl *et al*. [ 20 ], Benson *et al*. [ 21 ], and Sinnott *et al*. [ 22 ]. While, Primosch *et al*. [ 23 ], Galindo *et al*. [ 24 ] found no significant differences with buffered local anesthesia on faster onset of action. 

The duration of anesthesia in the present study was between 300-420 minutes in 50 patients of group I (8.4%) and patients in group II (7.5%) had duration of anesthesia between 60-240 minutes with a mean difference of 342.51 minutes for group I and 122.06 minutes for group II which showed a statistical significance of *P*<0.05 for group I. The above results had a similar association with the results stated by Afolabi *et al*. [ 25 ]. While, Christoph *et al*. [ 9 ] and Sinnott *et al*. [ 22 ] found no significant differences with the use of buffered lidocaine on the duration of action of anesthesia. This signifies that lidocaine when buffered with 8.4% sodium bicarbonate tends to prolong its action more than 3 hours, thereby improving pain reduction in patients postoperatively. 

The mechanism being, sodium bicarbonate when added to lidocaine increases the pH of the solution and produces carbon dioxide and water. The carbon dioxide once liberated eventually diffuses into the nerve membrane, decreases the pH at intercellular compartment, and converts RN to RNH+. Once the RNH molecules are produced, they fail to reconvert to RN because of its decreased pH level at the intercellular compartment. This traps the RHN molecules within the nerve membrane thereby prolonging the duration of the action of local anesthesia [ 26 ].

As the limitation of the study, the time duration between the first and second appointment for the removal of contralateral teeth was 10 days, which may be an attributing factor for the patients to experience pain in the second group. 

## Conclusion

The results of our study signify that 8.4 % buffered lidocaine hydrochloride was found to be superior and effective in the reduction of pain during injection with faster onset and longer duration of the action of local anesthesia. Although the use of buffered (8.4% and 7.5%) lidocaine hydrochloride is practical and inexpensive, 8.4% buffered lidocaine hydrochloride when compared with 7.5% buffered lidocaine hydrochloride significantly yielded a comfortable experience for the patients undergoing mandibular impacted third molar surgery.
